# Research progress on heteroterpene and meroterpenoid compounds from the *Rhododendron* genus and their NMR characterization and biological activity

**DOI:** 10.3389/fphar.2025.1584962

**Published:** 2025-07-29

**Authors:** Jingxin Mao, Meiyan Yang, Tingting Li, Yan Sun, Zhaoyue Dong, Honghong Zhan, Min Chen

**Affiliations:** ^1^Chongqing Key Laboratory of High Active Traditional Chinese Drug Delivery system, Chongqing Medical and Pharmaceutical College, Chongqing, China; ^2^Chongqing Key Laboratory of New Drug Screening from Traditional Chinese Medicine, Integrative Science Center of Germplasm Creation in Western China (Chongqing) Science City and Southwest University, Chongqing, China; ^3^SWU-TAAHC Medicinal Plant Joint R&D Centre, College of Pharmaceutical Sciences, Southwest University, Chongqing, China

**Keywords:** traditional Chinese medicines, *Rhododendron* genus, phytochemistry, biological activities, analyses

## Abstract

The plant genus, *Rhododendron* constitutes an important part of the treasure trove of traditional Chinese medicine and have made outstanding contributions to human health for centuries. There are approximately 25 species of *Rhododendron* plants in China that have been used in folk medicine. Among these, Dali, which is known as little *Rhododendron*, is one of the most commonly utilized species. Modern chemical and pharmacological studies have shown that the genus contains diverse chemical constituents, including terpenes, diterpenes, triterpenes, sesquiterpenes, monoterpenes of the resveratrol type, heteroterpenes, meroterpenoids, flavonoids, lignin, phenolic acids. Meroterpenoids are derived from terpenoid biogenic pathways, with a biosynthesis involving shikimic acid terpenoid adducts. Heteroterpenes, a class of terpenoids with diverse properties, are mainly derived from plants of the *Rhododendron* genus. This review manuscript collates 113 different terpenoid monomers identified in *Rhododendron* plants. Extracts of *Rhododendron* genus plants and purified terpenoid monomers exhibit numerous pharmacological effects, with anti-inflammatory, anticancer, analgesic, antibacterial, antioxidant, expectorant, anti-asthmatic, cough suppressant, and smooth muscle relaxation properties. The meroterpenoids and heteroterpenes have been shown to exhibit significant therapeutic effects in conditions such as ischemia-reperfusion injury and ischemic heart disease. The purpose of this article is to provide an overview of the chemical and pharmacological research on *Rhododendron* plants over the past 20 years, which may be of value in the development of new drugs or food supplements.

## 1 Introduction

*Rhododendron* is the largest genus in the *Ericaceae* family, and China boasts approximately 571 species of this genus, 409 of which are endemic. They are distributed throughout the country, except for the provinces of Xinjiang and Ningxia, but are predominantly concentrated in southwestern China in the regions of Tibet, Yunnan, and Sichuan (Liu et al., 2024; Hu, 2005; Yang et al., 2020; Fan et al., 2022). Among the most highly valued and prevalent landscape plants, most species in the *Rhododendron* genus exhibit exceptional ornamental qualities. Furthermore, approximately 25 species of *Rhododendron* in China have found application in traditional Chinese medicine and are extensively utilized in the treatment of acute and chronic tracheobronchitis, cough, rheumatism, rheumatoid arthritis, osteomyelitis, nephritis, venereal sores, abdominal pain, blood stagnation, menstrual irregularities, and various other ailments (Liu et al., 2024; Wang et al., 2010; Zreik, 2024; Heyadri et al., 2015; Fang et al., 2007).

Dari, also known as Paru or the Chinese name for little azalea, is primarily derived from the dried leaves and flowers of *R. cerasinum* Tagg (*Rhododendron primuliflorum*) and *R. anthopogonoides* Maxim, both belonging to the Ericaceae family. It is one of the most commonly used Tibetan medicines but, due to the different distribution areas of the two plants, there are regional differences in their application. Specifically, in Sichuan, Yunnan, and Tibetan traditional medicine, flowers of the cherry grass azalea *R. anthopogonoides* are used primarily to formulate medicines for internal use, while the leaves are generally utilized in medicinal baths to treat skin diseases. Conversely, Qinghai, Sichuan, and Tibetan doctors believe that both the leaves and flowers of *R. cerasinum* can be used internally (Beckwith, 1979; Reuter et al., 2013).

Dari is renowned for its properties of clearing heat, reducing swelling, and tonifying the kidneys. The Compendium of Materia Medica has systematically standardized the properties and functions of Dari: “Its leaves are bitter, astringent, and develop a bitter taste after digestion. Its efficacy includes treating cold stomach, improper diet, skin diseases, and stiffness of the limbs, with properties that are hot and sharp. Its flowers have a sweet, bitter, and astringent taste, which turns sweet after digestion. Their effects are light, hot, and dry, and they are primarily used to treat edema, water and soil disorders, lung diseases, bronchitis, weakness, and muteness.” (Popescu and Kopp, 2013; Qiang et al., 2011).

Among the medicinal plants of the *Rhododendron* genus, *R. molle* (Blume) G. Don has the longest history of use. The flowers of *R. molle* serve as the primary ingredient in hemp boiling powder, a renowned pain reliever and anesthetic (Zheng et al., 2024a). The earliest surviving classic medical monograph, the *Shennong Ben Cao Jing* (Classic of the Materia Medica of the Divine Husbandman), initially documented the flowers of *R. molle* as the “haunted goat’s flower,” highlighting their use in treating pain while cautioning that they were toxic (Jin et al., 2013). The 2020 edition of the Chinese Pharmacopoeia states that *R. molle* possesses a warm nature and a pungent flavor and is commonly utilized for the treatment of rheumatic arthralgia and pain (Xu et al., 2021). Clinically, the roots, flowers, and fruits of *R. molle* are employed in the treatment of rheumatoid arthritis, traumatic pain, scabies, severe hypertension, and supraventricular tachycardia (Emmerick, 1975; Yang et al., 2022; Luo et al., 2023; Zhou et al., 2017; Mei et al., 2023). It also presents insecticidal action, achieved through contact, fumigation, repellency, and growth inhibition (Zheng et al., 2024a).

Another commonly used folk medicine applies the branches and leaves of *R. micranthum* Turcz in the treatment of pain-related conditions like rheumatoid arthralgia, lumbago, post-partum arthralgia, dysentery, and bone fractures (Jin et al., 2021; Zhang et al., 2013; Sun et al., 2018; Zhou et al., 2020). Studies have revealed that the primary active constituents of *R. molle* and *R. micranthum* are wood veratridane-type diterpenes. These diterpenes are currently only known to exist in the *Rhododendron* genus (Zheng et al., 2024b; Cai et al., 2018; Li et al., 2013). With their complex and novel skeletal structures, as well as their extensive and remarkable biological activities, wood veratridine-type diterpenes have emerged as a focal point in phytochemical research on the *Rhododendron* genus.

The dried leaves of *R. dauricum L.*, possess a slightly pungent aroma and a bitter taste (Leong et al., 2020). They are known for their efficacy in alleviating cough and promoting expectoration of phlegm, and have traditionally been employed as a folk remedy for the treatment of acute and chronic bronchitis, asthma, hypertension, and coughing, among other conditions. *R. dauricum* is clinically utilized for the treatment of asthma and thick phlegm resulting from bronchitis, demonstrating remarkable efficacy. Nowadays, it is extensively incorporated into Chinese medicines, including Man Shan Hong Syrup and Man Shan Hong Capsule (Feng et al., 2023; Liang et al., 2023). Dari (Emmerick, 1975; Li et al., 2013; Leong et al., 2020), which is also utilized as an anti-inflammatory agent in Tibetan medicine for the treatment of rheumatoid arthritis and chronic bronchitis (Reuter et al., 2013; Yang et al., 2011; Shi et al., 2020; Liu et al., 2021). In addition, the flowers of *R. arboreum* Smith, commonly known in Tibet as Ta Ma, are efficacious in alleviating cough and asthma (Li, 2007). Furthermore, previous studies have reported that extracts from its petals possess the ability to inhibit the replication of severe acute respiratory syndrome coronavirus (SARS-CoV)-2 *in vitro* (Lingwan et al., 2023). *R. auriculatum* Hemsl. is also a widely recognized folk medicine whose bark and roots are utilized to treat coughs (Sun et al., 2019). Additionally, *R. decorum* Franch. is used externally for the treatment of bruises, rheumatism, and various pains (Zhu et al., 2018).

Modern pharmacological studies have demonstrated that *Rhododendron* species are abundant in diverse chemical constituents, including wood veratrylane-type diterpenes, heteroterpenes, triterpenes, flavonoids, lignans, phenolic acids, sesquiterpenes, and monoterpenes respectively (Beckwith, 1979; Popescu and Kopp, 2013; Qiang et al., 2011). They exhibit a broad spectrum of significant pharmacological effects, with particularly notable anti-inflammatory, analgesic, antibacterial, and proteintyrosine phosphatase-1B (PTP1B) inhibitory activities (Liu et al., 2024; Popescu and Kopp, 2013). Furthermore, previous research has indicated that their protective effect against ischemia-reperfusion injury offers a promising pathway for the treatment of ischemic heart disease and ischemic stroke (Jiang et al., 2023; Zhou et al., 2022), highlighting their significant potential both for research and in medicinal applications.

Various heteroterpene and meroterpenoid constituents have been identified in flowering *Rhododendrons*, including *R. dauricum* and *R. anthopogonoides*. These compounds have demonstrated a wide array of biological activities, including anti-inflammatory, PTP1B-inhibitory, anti-histamine-releasing, anti-human immunodeficiency virus (HIV), anti-herpes simplex virus (HSV)-1, *α*-glycosidase inhibition, and other properties (Liao et al., 2015; Zhang et al., 2022; Shi et al., 2020). In addition to their anti-acute kidney injury (AKI) effects in mice (Ashe and Zahs, 2010; Wu et al., 2021; Mizuta et al., 2009), other *in vivo* studies have demonstrated improvements in learning and memory ability, and anticoagulation in Alzheimer’s disease (AD) mice (Burchill et al., 2021; Jeon et al., 2022; Day et al., 2017; Wu et al., 2017). This comprehensive review aims to offer a summary of the chemical and pharmacological studies conducted on *Rhododendron* plants over the last 2 decades, potentially contributing to the advancement of novel drugs or food supplements.

## 2 An overview of the research on meroterpenoids and heteroterpenes in *Rhododendron* species

Meroterpenoids partially originate from the terpenoidogenic pathway and are biosynthesized through the combination of mangiferic acid and terpenoids (Pedraza-Chaverri et al., 2008; Jiang et al., 2002). This class of compounds, mainly found in algae, fungi, bacteria and some higher plants, shows a wide range of biological activities and chemical structures with unique backbones (Cornforth, 1968; Li et al., 2018; Zhao et al., 2017; Zhao et al., 2021). *Rhododendron* heteroterpenes are derivatives of chromane and chromene synthesized through a polyketide-terpene pathway.

Compounds **66** and **67** constitute the first pair of 6/6/6-ring heterobespene enantiomers to be isolated from *Rhododendron*, featuring a hexahydroxyanthracene moiety (Shi et al., 2020). Compounds **99** and **100** represent the first pair of heteromonoterpene enantiomers to feature a unique benzo [*b*]-2-oxa-[5.1.0]undecane 6/6/6/4-ring skeleton (Liao et al., 2015). Similarly, compounds **106** and **107** constitute the first pair of heteromonoterpene enantiomers with a benzo [*d*]-2,6-dioxa-tricyclo [5.2.2.0]undecane 6/6/6/5-ring system (Liao et al., 2017). Furthermore, compounds **112** and **113** are the second known pair of heteromonoterpene enantiomers to possess a benzo [*c*]-2,5,7-trioxa-tricyclo [7.2.1.0]dodecane 6/7/5/5-ring framework (Huang et al., 2018).

**FIGURE 1:**
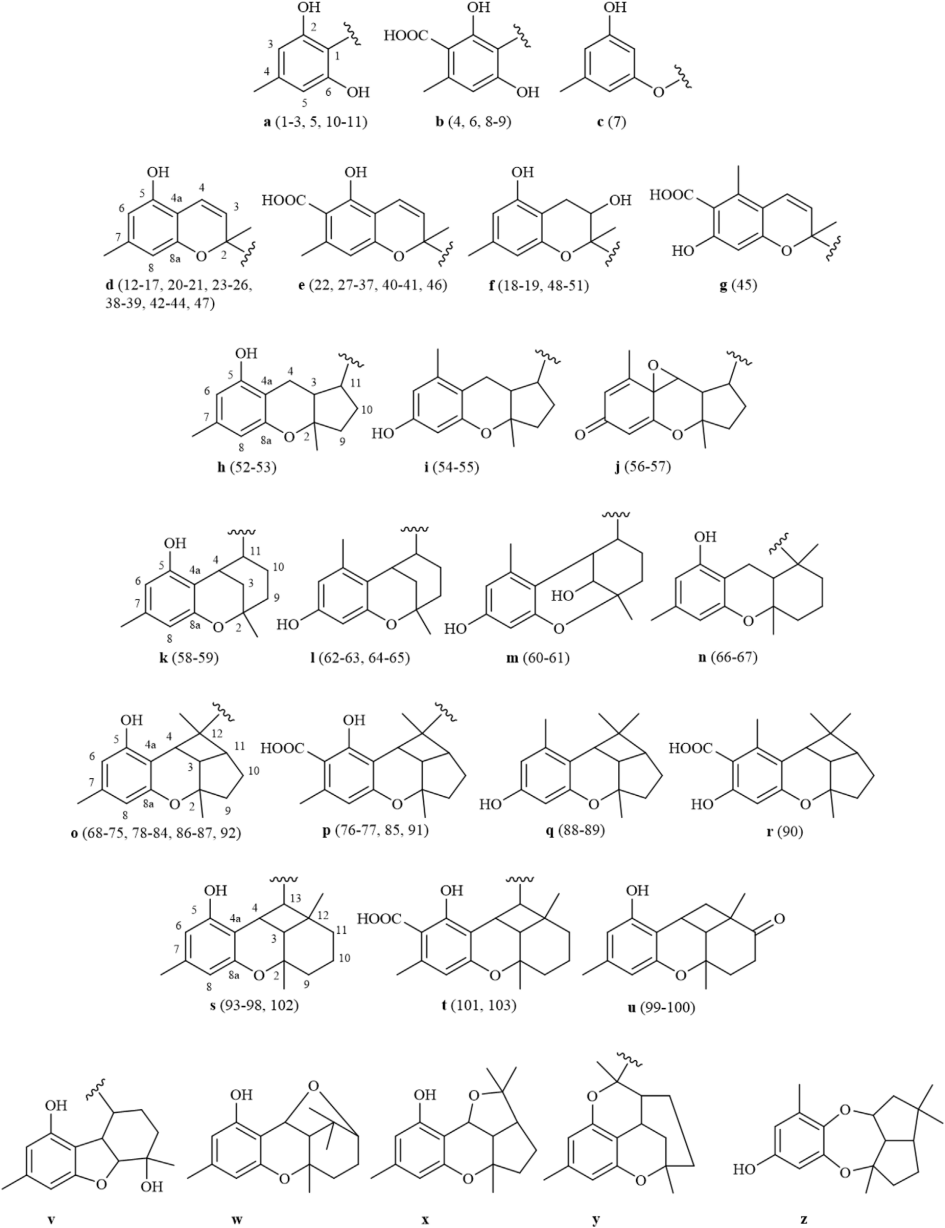
Structure types of heteroterpenes or meroterpenoids from genus *Rhododendron.*

Grifolin derivatives are considered as the significant precursors in the biosynthetic pathway of this class of compounds. Through cyclization and oxidation processes, these grifolin derivatives give rise to polycyclic heteroterpenes that exhibit complex and diverse skeletal structures (Shi et al., 2020; De Loose, 1969; Kashiwada et al., 2001). Furthermore, meroterpenoids of the *Rhododendron* genus primarily exist in the form of enantiomers, and heteroterpenoids from this genus encompass a variety of structural types, including bicyclic (**12**–**51**), 6/5/6-ring (**104**–**105**), 6/6/5-ring (**52**–**57**), 6/6/6-ring (**58**–**67**), 6/6/5/4-ring (**68**–**92**), and 6/6/6/4-ring (**93**–**103**) structures, among others (Figure 1). The bicyclic and 6/6/5/4-ring structures are the most prevalent among these.

Nuclear magnetic resonance (NMR) spectroscopy is a crucial and indispensable tool for elucidating the structure of compounds. The spectroscopic features of a particular class of chemical constituents can offer valuable guidance for their targeted separation and structural identification (Pedraza-Chaverri et al., 2008; Jiang et al., 2002). Therefore, a concise summary of the structures of meroterpenoids isolated from *Rhododendron* and their respective NMR spectroscopic characteristics is provided, aiming to establish a foundation for future research endeavors.

### 2.1 Structures of grifolin derivatives and their NMR spectroscopic characterization

**FIGURE 2:**
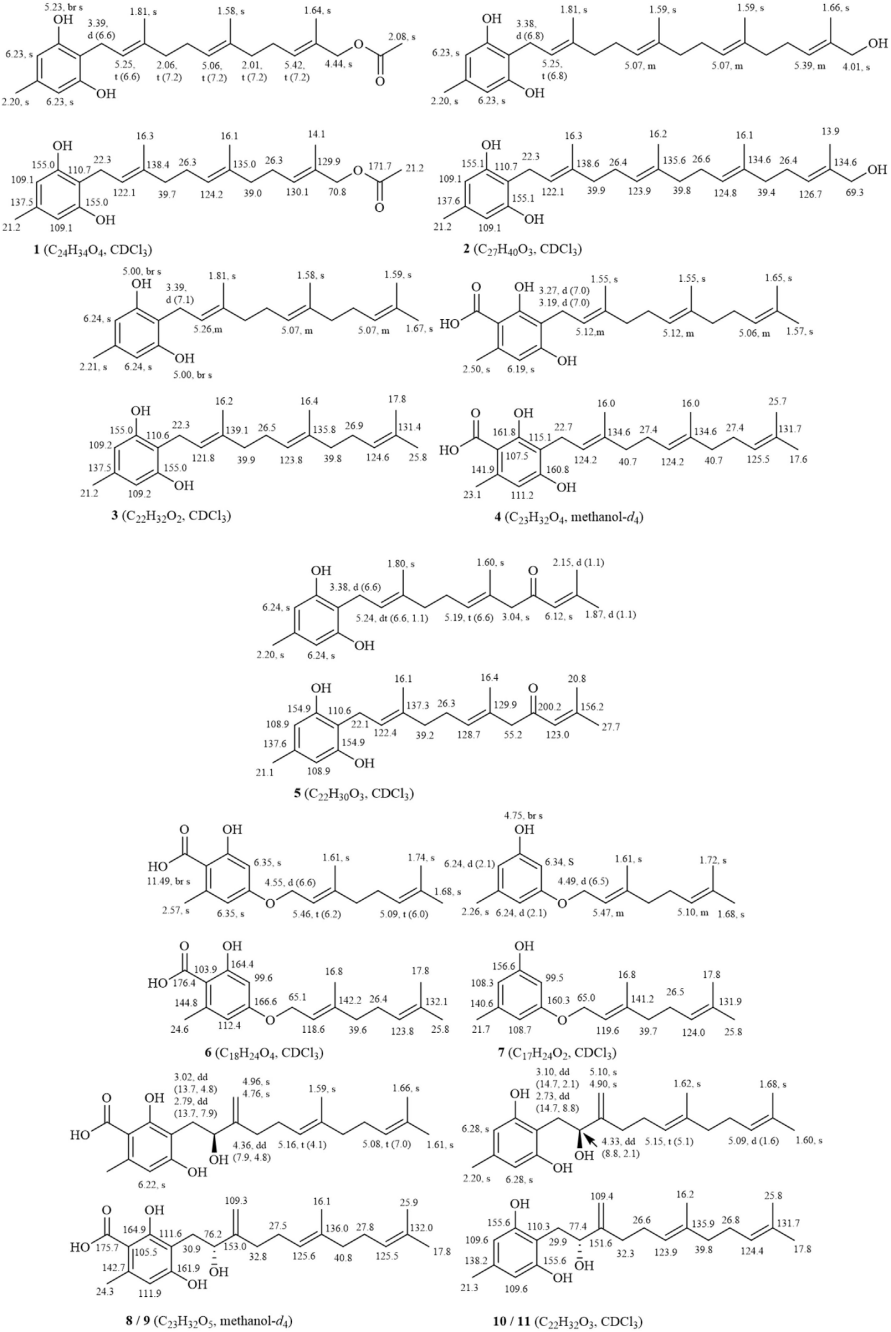
Structures and ^1^H-NMR/^13^C-NMR data of grifolin derivatives from genus *Rhododendron.*

The defining characteristic of the chemical structure of Grifolin derivatives (**1**–**11**) is a 1,2,3,5-tetrasubstituted benzene ring bearing a long single chain, frequently attached at the C-1 position. The side chain typically comprises two to four isopentenyl units. Compound **2**, which is the firstly heteroditerpene isolated from *Rhododendron* (Shi et al., 2020), possesses a side chain comprising four interconnected isopentenyl units, with a hydroxyl substituent on the methyl group of the final unit. Furthermore, some compounds feature a side chain with a 5-position hydroxyl group (**6** and **7**). When compounds exhibit a carboxyl group at the C-3 position (**4**, **6**, **8**, and **9**), the aryl ring engages in π-π conjugation with the -COOH group. This interaction leads to a shift in the electron cloud towards the more electronegative O atoms, causing the chemical shifts of the C-2, C-4, and C-6 positions to appear at lower field compared to those of the C-1, C-3, and C-5 positions that are relatively shielded. Consequently, the chemical shifts of their carbon spectra are typically 2.0–6.0 ppm lower than those observed in compounds lacking a C-3 carboxyl group (comparing **3** with **4**, **6** with **7**, and **8/9** with **10/11**). Without C-3 carboxyl substitution, a pair of magnetically equivalent aromatic proton signals typically appears in the low-field region of the ^1^H-NMR spectra for this class of compounds (**1**–**3**, **5**, **7**, **10**, and **11**). This characteristic, along with the presence of multiple isopentenyl signals, is commonly observed by ^1^H and ^13^C-NMR spectroscopy, serving as the primary method for distinguishing grifolin derivatives from other types of heteroterpene monomeric compounds. The names of the compounds, along with their plant sources and chemical structures, are provided in Table 1 and Figure 2.

**TABLE 1:** Grifolin derivatives from genus *Rhododendron*.

No.	Name	Plant source	Ref.
1	anthoponoid H	*R. anthopogonoides* Maxim. (twigs and leaves)	Shi et al. (2020)
2	anthoponoid I	*R. anthopogonoides* Maxim. (twigs and leaves)	Shi et al. (2020)
3	grifolin	*R. anthopogonoides* Maxim. (twigs and leaves)	Shi et al. (2020)
4	grifolic acid	*R. anthopogonoides* Maxim. (twigs and leaves)	Shi et al. (2020)
5	grifolinone A	*R. fastigiatum* Franch. (aerial parts)	Huang et al. (2019)
6	geranyl orsellinic acid	*R. anthopogonoides* Maxim. (twigs and leaves)	Iwata and Kitanaka (2011)
7	ranhuadujuanine D	*R. anthopogon* D. Don (leaves)	Qin et al. (2010)
8	(+)-nivalnoid C	*R. nivale* Hook. f. (twigs and leaves)	Zeng et al. (2023)
9	(−)-nivalnoid C	*R. nivale* Hook. f. (twigs and leaves)	Zeng et al. (2023)
10	(+)-nivalnoid D	*R. nivale* Hook. f. (twigs and leaves)	Zeng et al. (2023)
11	(−)-nivalnoid D	*R. nivale* Hook. f. (twigs and leaves)	Zeng et al. (2023)

### 2.2 Structures of bicyclic heteroterpenes or meroterpenoids and their NMR spectroscopic characterization

**FIGURE 3:**
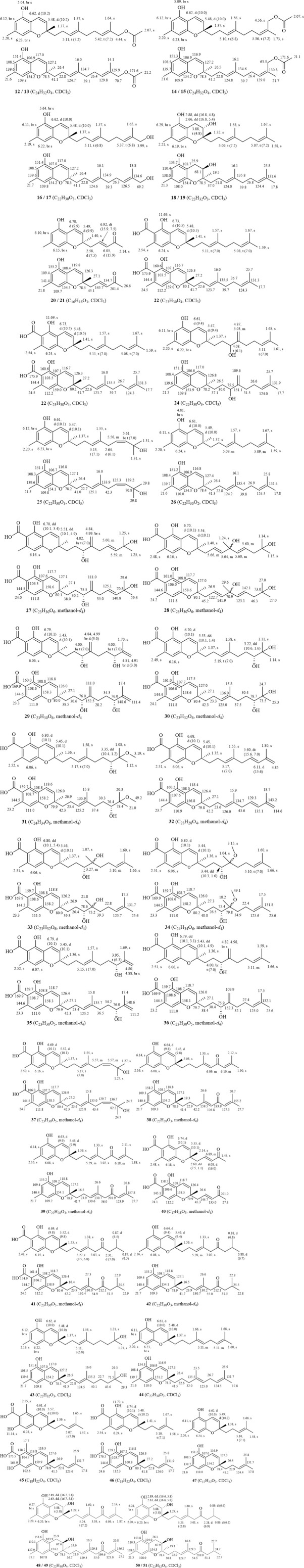
(1–3). Structures and ^1^H-NMR/^13^C-NMR data of bicyclic heteroterpenes or meroterpenoids from genus *Rhododendron.*

When there is no substituent at the C-6 position, bicyclic heteroterpenes or meroterpenoids (**12**–**51**) typically exhibit a sequential pattern of double-single-single-double peak signals in their ^1^H-NMR spectra in the range 5.40–6.80 ppm. This pattern consists of a set of cis-coupled olefinic proton signals and two aromatic proton signals (δ_H_: 6.10–6.30 ppm). This feature is primarily utilized to distinguish bicyclic heteroterpenes with a double bond at the 3,4-position from other types of heteroterpenes. Typically, the chemical shift values for H-4 are around 6.60–6.80 ppm, and for H-3, they are approximately 5.40–5.50 ppm. Additionally, the coupling constant value J is generally around 10.0 Hz. Furthermore, a small number of bicyclic heteroterpenes undergo addition reactions at the 3,4-positions, particularly when there is hydroxyl substitution at the C-3 position (**18**–**19** and **48**–**51**). In such cases, H-3 typically appears as a triplet (t-peak) with a chemical shift value of approximately 3.88 ppm, while H_2_-4 exhibits a doublet of doublets (dd-peak) with chemical shift values of around 2.89 ppm and 2.63 ppm. Certain bicyclic meroterpenoids frequently undergo substitution at the C-6 position by a carboxyl group, and their carbon spectral chemical shift values are similar to those observed in grifolin derivatives (**21/40**, **41/42**, and **46/47**). The C-9 position of bicyclic meroterpenoids is commonly linked to a side chain comprising either one or two isopentenyl units. The absolute configuration of the 2-position in bicyclic meroterpenoids can be deduced using the chromane/chromene helix rule (Liang et al., 2021; Presley et al., 2018; Górecki et al., 2014; Makoto et al., 2008; Lin et al., 2015). The names, source plants, and chemical structures of bicyclic meroterpenoids are presented in Table 2 and Figures 1–3, **3-2**, and **3-3**.

**TABLE 2:** Bicyclic heteroterpenes or meroterpenoids from genus *Rhododendron*.

No.	Name	Plant source	Ref.
12	(+)-anthoponoid E	*R. anthopogonoides* Maxim. (twigs and leaves)	Shi et al. (2020)
13	(−)-anthoponoid E	*R. anthopogonoides* Maxim. (twigs and leaves)	Shi et al. (2020)
14	(+)-anthoponoid F	*R. anthopogonoides* Maxim. (twigs and leaves)	Shi et al. (2020)
15	(−)-anthoponoid F	*R. anthopogonoides* Maxim. (twigs and leaves)	Shi et al. (2020)
16	(+)-daurichromene D	*R. anthopogonoides* Maxim. (twigs and leaves)	Shi et al. (2020)
17	(−)-daurichromene D	*R. anthopogonoides* Maxim. (twigs and leaves)	Shi et al. (2020)
18	(+)-anthoponoid G	*R. anthopogonoides* Maxim. (twigs and leaves)	Shi et al. (2020)
19	(−)-anthoponoid G	*R. anthopogonoides* Maxim. (twigs and leaves)	Shi et al. (2020)
20	(−)-rubiginosin H	*R. dauricum* L. (twigs and leaves)	Zhang et al. (2022)
21	(+)-rubiginosin H	*R. dauricum* L. (twigs and leaves)	Zhang et al. (2022)
22	daurichromenic acid	*R. dauricum* L. (twigs and leaves)	Zhao et al. (2021)
23	daurichromene A	*R. dauricum* L. (twigs and leaves)	Iwata et al. (2004)
24	daurichromene B	*R. dauricum* L. (twigs and leaves)	Iwata et al. (2004)
25	daurichromene C	*R. dauricum* L. (twigs and leaves)	Iwata et al. (2004)
26	confluentin	*R. dauricum* L. (twigs and leaves)	Iwata et al. (2004)
27	capitachromenic acid A	*R. capitatum* Maxim. (aerial parts)	Liang et al. (2021)
28	capitachromenic acid B	*R. capitatum* Maxim. (aerial parts)	Liang et al. (2021)
29	capitachromenic acid C	*R. capitatum* Maxim. (aerial parts)	Liang et al. (2021)
30	capitachromenic acid D	*R. capitatum* Maxim. (aerial parts)	Liang et al. (2021)
31	capitachromenic acid G	*R. capitatum* Maxim. (aerial parts)	Liang et al. (2021)
32	capitachromenic acid H	*R. capitatum* Maxim. (aerial parts)	Liang et al. (2021)
33	capitachromenic acid E	*R. capitatum* Maxim. (aerial parts)	Liang et al. (2021)
34	capitachromenic acid K	*R. capitatum* Maxim. (aerial parts)	Liang et al. (2021)
35	capitachromenic acid I	*R. capitatum* Maxim. (aerial parts)	Liang et al. (2021)
36	capitachromenic acid J	*R. capitatum* Maxim. (aerial parts)	Liang et al. (2021)
37	capitachromenic acid M	*R. capitatum* Maxim. (aerial parts)	Liang et al. (2021)
38	rubiginosin D	*R. rubiginosum* Franch. var. rubiginosum (flowers)	Yang et al. (2018)
39	rubiginosin E	*R. rubiginosum* Franch. var. rubiginosum (flowers)	Yang et al. (2018)
40	rubiginosin F	*R. rubiginosum* Franch. var. rubiginosum (flowers)	Yang et al. (2018)
41	anthopogochromene A	*R. rubiginosum* Franch. var. rubiginosum (flowers)	Yang et al. (2018)
42	anthopogochromene B	*R. rubiginosum* Franch. var. rubiginosum (flowers)	Yang et al. (2018)
43	rhodomeroterpene	*Rhododendron* genus	Wu et al. (2021)
44	isoconfluentin	*R. rubiginosum* Franch. var. rubiginosum (leaves)	Mizuta et al. (2009)
45	anthopogochromenic acid	*R. anthopogonoides* Maxim. (twigs and leaves)	Iwata and Kitanaka (2011)
46	cannabiorcichromenic acid	*R. anthopogonoides* Maxim. (twigs and leaves)	Iwata and Kitanaka (2011)
47	2,7-dimethyl-2-(4-methylpent-3-enyl)-3,4-dihydrochromen-5-ol	*R. anthopogonoides* Maxim. (twigs and leaves)	Iwata and Kitanaka (2011)
48	(+)-nivalone A	*R. nivale* Hook. f. (twigs and leaves)	Zeng et al. (2023)
49	(−)-nivalone A	*R. nivale* Hook. f. (twigs and leaves)	Zeng et al. (2023)
50	(+)-nivalone B	*R. nivale* Hook. f. (twigs and leaves)	Zeng et al. (2023)
51	(−)-nivalone B	*R. nivale* Hook. f. (twigs and leaves)	Zeng et al. (2023)

### 2.3 Structures of polycyclic heteroterpenes or meroterpenoids and their NMR spectroscopic characterization

Polycyclic heteroterpenes or meroterpenoids have been reported predominantly to possess a 6/6/5/4-ring skeleton (**68**–**92**). The chemical shift values for H-3 in this type of compound range from 2.50 to 2.70 ppm and are displayed as dd peaks in the ^1^H-NMR spectra. The J-values associated with these peaks are in the ranges 9.0–10.0 Hz and 7.0–8.0 Hz. The chemical shift values for the H-4 position range between 3.00 and 3.30 ppm, exhibiting d-peaks with J-values in the range 9.0–10.0 Hz. Conversely, the chemical shift values for H-11 lie between 2.40 and 2.50 ppm, displaying td-peaks. The proton signals of the 6/6/5/4-ring meroterpenoids, specifically H-3, H-4, and H-11, serve as distinguishing features that differentiate them from other heteroterpenes. Additionally, the values of the coupling constants between H-3 and H-4 (*J*_3-4_) and between H-3 and H-11 (*J*_3-11_) can be utilized in conjunction with the rotating frame overhauser effect spectroscopy (ROESY) spectra of the compounds to determine their relative configuration (Shi et al., 2020; Zhao et al., 2021; Huang et al., 2018; Huang et al., 2019). In the 6/6/5/4-ring heteroterpenes derived from *Rhododendron*, H-3, H-4, and H-11 typically reside on the same side of the plane. Furthermore, compounds of this type generally possess a side chain comprising an isopentenyl unit attached at the C-13 position.

**FIGURE 4:**
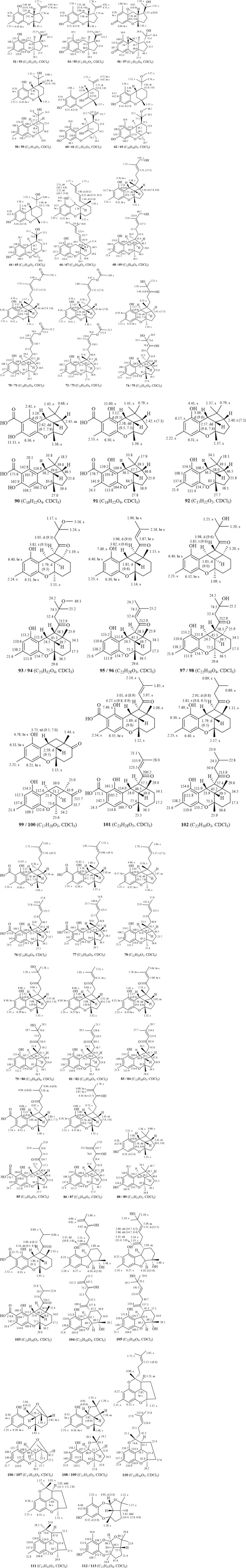
(1–4). Structures and ^1^H-NMR/^13^C-NMR data of polycyclic heteroterpenes or meroterpenoids from genus *Rhododendron.*

A total of eleven 6/6/6/4-ring heteroterpene monomers (**93**–**103**) have been reported in *Rhododendron*. In comparison to the 6/6/5/4-ring heteroterpenes, this type of compound exhibits a d-peak for H-3, with a chemical shift in the range 1.80–1.90 ppm, and a t-peak for H-4, typically showing a chemical shift at 3.80–4.30 ppm. H-13 displays a d-peak with chemical shift values in the range 2.90–4.00 ppm and J-values between 9.0 and 10.0 Hz. Notably, the hydrogen signals of H-3 and H-4 are the primary features that differentiate these compounds from 6/6/5/4-ring cycloheteroterpenoids. The values of the coupling constants between H-3 and H-4 (*J*_3-4_) can be utilized in conjunction with ROESY spectra to ascertain the relative configuration of the compounds. Additionally, in the compounds reported to date (Zhang et al., 2022), H-3 and H-4 are positioned on the same side of the plane. For meroterpenoid 6/6/6-ring compounds (**58–65**), H-4 chemical shifts range from 3.20–3.50 ppm and typically appear as t-peaks or broad singlets (br s). Coupling constants *J*_4-11_ (between H-4 and H-11), rather than *J*_3-4_, can determine relative configuration (Huang et al., 2018). Compounds of this type may feature a C-3 hydroxyl group (**60**–**61**) and, when determining their relative configuration, it is often necessary to consider the influence of the *γ*-gauche effect arising from this 3-OH. In such cases, the values of the coupling constants between H-3 and H-4 (*J*_3-4_) can serve as a reference for establishing the relative configuration (Huang et al., 2018; Wang et al., 2017). Furthermore, the 6/6/5- and 6/6/6-ring heteroterpenes frequently undergo a positional shift of the aromatic methyl group and the 5-OH (**52/53**, **54/55**, **58/59**, and **64/65**). Similarly, the 6/6/5/4-ring heteroterpenes exhibit the same phenomenon when lacking side-chain substitution at the C-13 position (**88/89** and **92**, as well as **90** and **91**). The names, source plants, and chemical structures of the polycyclic heteroterpenes are presented in Table 3 and Figures 1–4, **4–2**, **4-3**, and **4-4**.

**TABLE 3:** Polycyclic heteroterpenes or meroterpenoids from genus *Rhododendron*.

No.	Name	Plant source	Ref.
52	(−)-fastinoid D	*R. fastigiatum* Franch. (aerial parts)	Huang et al. (2019)
53	(+)-fastinoid D	*R. fastigiatum* Franch. (aerial parts)	Huang et al. (2019)
54	(−)-nyingchinoid C	*R. nyingchiense* R. C. Fang and S. H. Huang (aerial parts)	Huang et al. (2018)
55	(+)-nyingchinoid C	*R. nyingchiense* R. C. Fang and S. H. Huang (aerial parts)	Huang et al. (2018)
56	(+)-nyingchinoid B	*R. nyingchiense* R. C. Fang and S. H. Huang (aerial parts)	Huang et al. (2018)
57	(−)-nyingchinoid B	*R. nyingchiense* R. C. Fang and S. H. Huang (aerial parts)	Huang et al. (2018)
58	(−)-rhodonoid G	*R. capitatum* Maxim. (aerial parts)	Liao et al. (2017)
59	(+)-rhodonoid G	*R. capitatum* Maxim. (aerial parts)	Liao et al. (2017)
60	(+)-nyingchinoid E	*R. nyingchiense* R. C. Fang and S. H. Huang (aerial parts)	Huang et al. (2018)
61	(−)-nyingchinoid E	*R. nyingchiense* R. C. Fang and S. H. Huang (aerial parts)	Huang et al. (2018)
62	(+)-nyingchinoid F	*R. nyingchiense* R. C. Fang and S. H. Huang (aerial parts)	Huang et al. (2018)
63	(−)-nyingchinoid F	*R. nyingchiense* R. C. Fang and S. H. Huang (aerial parts)	Huang et al. (2018)
64	(+)-nyingchinoid G	*R. nyingchiense* R. C. Fang and S. H. Huang (aerial parts)	Huang et al. (2018)
65	(−)-nyingchinoid G	*R. nyingchiense* R. C. Fang and S. H. Huang (aerial parts)	Huang et al. (2018)
66	(−)-anthoponoid A	*R. anthopogonoides* Maxim. (twigs and leaves)	Shi et al. (2020)
67	(+)-anthoponoid A	*R. anthopogonoides* Maxim. (twigs and leaves)	Shi et al. (2020)
68	(−)-anthoponoid B	*R. anthopogonoides* Maxim. (twigs and leaves)	Shi et al. (2020)
69	(+)-anthoponoid B	*R. anthopogonoides* Maxim. (twigs and leaves)	Shi et al. (2020)
70	(−)-anthoponoid C	*R. anthopogonoides* Maxim. (twigs and leaves)	Shi et al. (2020)
71	(+)-anthoponoid C	*R. anthopogonoides* Maxim. (twigs and leaves)	Shi et al. (2020)
72	(+)-anthoponoid D	*R. anthopogonoides* Maxim. (twigs and leaves)	Shi et al. (2020)
73	(−)-anthoponoid D	*R. anthopogonoides* Maxim. (twigs and leaves)	Shi et al. (2020)
74	(−)-rhodonoid I	*R. dauricum* L. (twigs and leaves)	Zhang et al. (2022)
75	(+)-rhodonoid I	*R. dauricum* L. (twigs and leaves)	Zhang et al. (2022)
76	rhododaurichromanic acid A	*R. dauricum* L. (twigs and leaves)	Zhao et al. (2021)
77	rhododaurichromanic acid B	*R. dauricum* L. (twigs and leaves)	Zhao et al. (2021)
78	*rel*-(1*R*,1a*S*,3a*R*,8b*R*,8c*R*)-1a,2,3,3a,8b,8c-hexahydro-1,3a,6-trimethyl-1-(4-methyl-3-penten-1-yl)-1 *H*-4-oxabenzo [*f*]cyclobut [*cd*]inden-8-ol	*R. dauricum* L. (leaves)	Martiningtiyas et al. (2024)
79	(+)-fastinoid A	*R. fastigiatum* Franch. (aerial parts)	Huang et al. (2019)
80	(−)-fastinoid A	*R. fastigiatum* Franch. (aerial parts)	Huang et al. (2019)
81	(+)-fastinoid B	*R. fastigiatum* Franch. (aerial parts)	Huang et al. (2019)
82	(−)-fastinoid B	*R. fastigiatum* Franch. (aerial parts)	Huang et al. (2019)
83	(−)-rhodonoid B	*R. capitatum* Maxim. (aerial parts)	Liao et al. (2015)
84	(+)-rhodonoid B	*R. capitatum* Maxim. (aerial parts)	Liao et al. (2015)
85	rubiginosin C	*R. rubiginosum* Franch. var. rubiginosum (flowers)	Yang et al. (2018)
86	(+)-rhodonoid E	*R. capitatum* Maxim. (aerial parts)	Liao et al. (2017)
87	(−)-rhodonoid E	*R. capitatum* Maxim. (aerial parts)	Liao et al. (2017)
88	(−)-nyingchinoid D	*R. nyingchiense* R. C. Fang and S. H. Huang (aerial parts)	Huang et al. (2018)
89	(+)-nyingchinoid D	*R. nyingchiense* R. C. Fang and S. H. Huang (aerial parts)	Huang et al. (2018)
90	anthopogocyclolic acid	*R. anthopogonoides* Maxim. (twigs and leaves)	Iwata and Kitanaka (2011)
91	cannabiorcicyclolic acid	*R. anthopogonoides* Maxim. (twigs and leaves)	Iwata and Kitanaka (2011)
92	ranhuadujuanine A	*R. anthopogonoides* Maxim. (twigs and leaves)	Iwata and Kitanaka (2011)
93	(−)-rhodonoid H	*R. dauricum* L. (twigs and leaves)	Zhang et al. (2022)
94	(+)-rhodonoid H	*R. dauricum* L. (twigs and leaves)	Zhang et al. (2022)
95	(−)-rubiginosin A	*R. dauricum* L. (aerial parts)	Huang et al. (2019)
96	(+)-rubiginosin A	*R. dauricum* L. (aerial parts)	Huang et al. (2019)
97	(−)-fastinoid C	*R. dauricum* L. (aerial parts)	Huang et al. (2019)
98	(+)-fastinoid C	*R. dauricum* L. (aerial parts)	Huang et al. (2019)
99	(−)-rhodonoid A	*R. capitatum* Maxim. (aerial parts)	Liao et al. (2015)
100	(+)-rhodonoid A	*R. capitatum* Maxim. (aerial parts)	Liao et al. (2015)
101	rubiginosin B	*R. rubiginosum* Franch. var. rubiginosum (flowers)	Yang et al. (2018)
102	rubiginosin G	*R. rubiginosum* Franch. var. rubiginosum (flowers)	Yang et al. (2018)
103	anthopogochromane	*R. anthopogonoides* Maxim. (twigs and leaves)	Iwata and Kitanaka (2010)
104	ferruginene A	*R. ferrugineum* L. (leaves)	Seephonkai et al. (2011)
105	ferruginene B	*R. ferrugineum* L. (leaves)	Seephonkai et al. (2011)
106	(+)-rhodonoid C	*R. capitatum* Maxim. (aerial parts)	Liao et al. (2017)
107	(−)-rhodonoid C	*R. capitatum* Maxim. (aerial parts)	Liao et al. (2017)
108	(−)-rhodonoid D	*R. capitatum* Maxim. (aerial parts)	Liao et al. (2017)
109	(+)-rhodonoid D	*R. capitatum* Maxim. (aerial parts)	Liao et al. (2017)
110	*rel*-(6*R*,6a*S*,9*R*,10a*R*)-6a,7,8,9,10,10a-hexahydro-3,6,9-trimethyl-6-(4-methyl-3-penten-1-yl)-1,9-epoxy-6*H*-dibenzo [*b,d*]pyran	*R. dauricum* L. (leaves)	Martiningtiyas et al. (2024)
111	ranhuadujuanine B	*R. anthopogon* D. Don (leaves)	Qin et al. (2010)
112	(−)-nyingchinoid A	*R. nyingchiense* R. C. Fang and S. H. Huang (aerial parts)	Huang et al. (2018)
113	(+)-nyingchinoid A	*R. nyingchiense* R. C. Fang and S. H. Huang (aerial parts)	Huang et al. (2018)

## 3 Overview of the biological activities of heteroterpenes or meroterpenoids in the *Rhododendron* genus

Most of the heteroterpenes or meroterpenoids isolated from *Rhododendron* have been reported to exhibit a wide range of bioactivities, including anti-inflammatory, PTP1B inhibitory, antihistamine-releasing, anti-HIV, anti-herpes simplex virus (HSV-1), anti-tumor, and *α*-glucosidase inhibitory activities. The anti-inflammatory and PTP1B-inhibitory activities are particularly significant among these (Liang et al., 2021). Hou et al. (Wu et al., 2021) discovered that the novel compound rhodomeroterpene (**43**) exhibited ameliorative effects in various models of renal injury. Pretreatment with this compound (30 mg/kg/d, ip, 3d) significantly suppressed the acute inflammatory response in LPS-induced septic mice. The mechanism of action may involve the regulation of inflammatory signaling pathways, such as IKK/NF-*к*B and PI3K/PDK1/Akt, especially in macrophages. This study indicates that rhodomeroterpene holds potential as a lead compound for the treatment of acute kidney injury (AKI).

Yang et al. reported that isoconfluentin (**44**), a novel bicyclic heteroterpenoid compound isolated from *R. nivale* Hook.f., exhibited significant anticoagulant effects in a dose-dependent manner (Mizuta et al., 2009). The compounds (+)-nivalnoid C (**8**) and (+)-nivalone B (**50**) isolated from *R. nivale* demonstrated protective effects against oxidative damage in nerve cells (Zeng et al., 2023). Xu et al. screened for the activity of heteroterpenoid cannabichromeorcinic acid (CA) from *R. primuliflorum*, finding anti-acetylcholinesterase activity (Iwata and Kitanaka, 2011). This compound significantly improved the memory and learning ability of mice and had a notable inhibitory effect on acetylcholinesterase in the brain tissue and serum of mice with Alzheimer’s disease (AD). Therefore, CA can be considered a promising lead compound for the development of therapeutic drugs for AD. Tables 1–4 present the significant active meroterpenoids derived from the genus *Rhododendron*. Based on this literature review, it appears that grifolin derivatives and bicyclic meroterpenoids exhibit superior anti-inflammatory, PTP1B, and *α*-glycosidase inhibitory activities. However, there are relatively few studies on the bioactivity of this class of constituents, and the available experimental data is insufficient to draw definitive conclusions.

**TABLE 4:** The activity of heteroterpenes or meroterpenoids from genus *Rhododendron in vitro*.

Compound	Activity	Model	Posive control	Results	Ref.
grifolin (3)	PTP1B inhibition	PTP1B	Oleanolic acid IC_50_ = 2.5 ± 0.2 *μ*M	IC_50_ = 5.7 ± 0.5 *μ*M	Huang et al. (2018)
grifolic acid (4)	PTP1B inhibition	PTP1B	PK-682 IC_50_ = 6.9 ± 0.2 *μ*M	IC_50_ = 3.6 ± 0.1 *µ*M	Liang et al. (2021)
grifolic acid (4)	Alpha-glucosidase inhibition	Alpha-glucosidase	Acarbose IC_50_ = 796.2 ± 73.2 *μ*M	IC_50_ = 8.0 ± 0.3 *µ*M	Liang et al. (2021)
(+)-rubiginosin H (21)	Anticancer	LPS induces NO release from RAW 264.7 cells	Dexamethasone	IC_50_ = 6.9 ± 0.97 *µ*M	(Zhang et al., 2022)
daurichromenic acid (22)	Anti-HIV	HIV-1-infected H9 cells	Zidovudine (AZT)	EC_50_ = 0.00567 *μ*g/mL; TI = 3710	Zhao et al. (2021)
daurichromenic acid (22)	Alpha-glucosidase inhibition	Alpha-glucosidase	Acarbose	IC_50_ = 10.6 ± 0.3 *µ*M	Liang et al. (2021)
daurichromenic acid (22)	PTP1B inhibition	PTP1B	PK-682 IC_50_ = 6.9 ± 0.2 *μ*M	IC_50_ = 2.5 ± 0.2 *µ*M	Liang et al. (2021)
capitachromenic acid C (29)	Alpha-glucosidase inhibition	Alpha-glucosidase	Acarbose IC_50_ = 796.2 ± 73.2 *μ*M	IC_50_ = 93.5 ± 2.3 *µ*M	Liang et al. (2021)
capitachromenic acid I (35)	Alpha-glucosidase inhibition	Alpha-glucosidase	Acarbose IC_50_ = 796.2 ± 73.2 *μ*M	IC_50_ = 21.2 ± 1.4 *µ*M	Liang et al. (2021)
capitachromenic acid J (36)	Alpha-glucosidase inhibition	Alpha-glucosidase	Acarbose IC_50_ = 796.2 ± 73.2 *μ*M	IC_50_ = 18.6 ± 0.7 *µ*M	(Liang et al., 2021)
capitachromenic acid J (36)	PTP1B inhibition	PTP1B	PK-682 IC_50_ = 6.9 ± 0.2 *μ*M	IC_50_ = 6.0 ± 0.6 *µ*M	Liang et al. (2021)
anthopogochromenic acid (45)	Antihistamine release	Histamine release from rat peritoneal mast cells	indomethacin IC_50_ = 250 *µ*M	IC_50_ = 64 *μ*M	Iwata and Kitanaka (2011)
2,7-dimethyl-2-(4-methylpent-3-enyl)-3,4-dihydrochromen-5-ol (47)	Antihistamine release	Histamine release from rat peritoneal mast cells	Indomethacin IC_50_ = 250 *µ*M	IC_50_ = 87 *μ*M	Iwata and Kitanaka (2011)
rhododaurichromanic acid A (76)	anti-HIV	HIV-1-infected H9 cells	AZT	CE_50_ = 0.37 *μ*g/mL TI = 91.9	Zhao et al. (2021)

## 4 Discussion

*Rhododendron* are quintessential alpine plants. As dominant or constructive species, they give rise to characteristic scrub communities in subtropical regions. These plants are predominantly distributed across the alpine and subalpine tree-line areas, as well as regions above the tree line, in western and southwestern China (Ștefănescu et al., 2019). They exert a substantial influence on various aspects, including the climate-change response of their distribution areas, the biogeochemical cycle, and the sustainable livelihoods of mountain-dwelling communities (Luo, 2020). *Rhododendron* possess a branching structure and are firmly rooted. The taxa that thrive in the alpine zone hold significant ecological importance, not only for water conservation and the maintenance of inter-regional hydrothermal balance, but also for defining the distinctive functions and services of mountain ecosystems, as well as contributing to biodiversity (Tomoki et al., 2019). The principal chemical constituents of *Rhododendron* encompass flavonoids, diterpenoids, triterpenoids, phenols, tannins, and volatile oils, among others. These compounds exhibit a range of pharmacological properties, including expectorant, cough-suppressant, anti-rheumatoid arthritis, anti-chronic bronchitis, cardiovascular-disease-treating, neuro-regulatory, anti-inflammatory, analgesic, stomachic, decongestant, and immune-modulating effects (Quang et al., 2006). Given their high medicinal value, the application of *Rhododendron* in the field of healthcare holds significant untapped potential. Volatile oils are extracted from aromatic plant materials. For instance, the leaves and flowers of the strongly-scented azalea, along with the leaves and shoots of the beauty azalea, are particularly rich in volatile oils. Additionally, these plant parts also contain ellagitannic substances, from which valuable extracts can be derived (Nukata et al., 2002). Furthermore, *Rhododendron* serve as a common source of animal fodder. In certain ethnic-populated regions, the local inhabitants still maintain the practice of consuming rhododendron crowns (Mao et al., 2017). Additionally, some species of *Rhododendron* boast dense, finely-grained wood, which makes them highly suitable for craft-making purposes. The aforementioned points collectively illustrate that the diversity of ecosystem functions and services provided by *Rhododendron* remains a central focus of both national and international research endeavors (Srivastava, 2012).

Currently, the taxonomic study of the genus *Rhododendron* has embraced modern technological advancements, enabling researchers to largely identify its related species. However, the majority of existing classification efforts for this genus are confined to specific major classes or subgenera within a given class (Dampc and Luczkiewicz, 2013). Furthermore, there is an absence of scientifically sound and standardized criteria for species classification, as well as uniform nomenclature guidelines. Additionally, the existing classification systems lack a comprehensive corresponding collection of specimens (Cross, 1975). Now, there exists a dearth of more systematic investigations into the relationship between the chemical constituents and pharmacological activities of *Rhododendron* species (Chamberlain et al., 1996). Consequently, research on their pharmacological effects requires further enhancement. The pharmacological mechanisms of action underlying the active ingredients in these species remain unclear and warrant in-depth exploration (Liu et al., 2024; Vengrytė and Raudonė, 2024; Sun et al., 2024; Zhang et al., 2024). Moreover, *Rhododendron* species have received relatively limited attention with regard to the establishment of quality standards (Yu et al., 2024; Kukhtenko et al., 2024; Mangral et al., 2025).

## 5 Conclusion

The genus *Rhododendron* is abundant in phytochemical resources, with numerous medicinal plants that exhibit significant anti-inflammatory and analgesic properties with great potential therapeutic value (Islam et al., 2023a). Furthermore, genus-specific heteroterpene/meroterpenoid constituents show significant activities in anti-inflammation, anti-histamine release, PTP1B inhibition, and α-glucosidase inhibition. The genus has also been applied in the emerging field of cyberpharmacology (Islam et al., 2023b). Meroterpenoids and heteroterpenes are secondary metabolites with structures partially derived from terpenoid pathways, and are research hotspots in the study of the chemical composition of *Rhododendron* species (Gu et al., 2025). To date, 113 monomers with diverse ring system skeletons and a wide variety of structural types have been isolated in *Rhododendron*. Among them, bicyclic and 6/6/5/4-ring heteroterpenes are predominant (Pan et al., 2023). These compounds may be differentiated based on the characteristics of their ^1^H-NMR spectra. However, most of the reported compounds are enantiomers obtained through chiral column separation of the racemate (Lin, 2024). Research on this class of constituents is insufficient, highlighting the need for further studies on the chemical composition of *Rhododendron* with the aim of discovering heteroterpene or meroterpenoid constituents with novel skeletal structures and significant pharmacological activities (Shi et al., 2020). This will provide potential active lead compounds for the research and development of new drugs, and also offer a reference for the development and application of *Rhododendron*-related medicinal plants (Li et al., 2008; Jing et al., 2015). In addition, by combining computer methods in the future, innovative momentum can be injected into the research of natural medicine chemistry and pharmacology from multiple dimensions (Sang et al., 2024; Iwata and Kitanaka, 2010; Nakatsuka et al., 2005; Wei et al., 2023). Through data integration, model construction, and intelligent analysis, the dual improvement of research efficiency and accuracy can be achieved.
